# Infectious pathogens associated with stillbirth and under-five mortality by sickle cell disease in Western Kenya: a cross-sectional study

**DOI:** 10.1186/s12879-026-13848-9

**Published:** 2026-06-30

**Authors:** Morgan Otundo Wasilwa, Valentine Simiyu, Fredrick Onduru, Edwin Kiplelgo, Maryann Nyanjom, Kephas Otieno, Hellen Muttai, Richard Omore, Victor Akelo, Dorcas Obiri-Yeboah

**Affiliations:** 1 Department of Microbiology and Immunology, University of Cape Coast, Cape Coast, Ghana; 2 Kenya Medical Research Institute-Centre for Global Health Research, Kisumu, Kenya; 3 Liverpool School of Tropical Medicine, Liverpool, UK

**Keywords:** Infectious pathogens, Stillbirth, Under-five mortality, Sickle cell disease, LMICs

## Abstract

**Background:**

Infectious Pathogens (IP) remain a significant contributor to stillbirths and under-five deaths in resource-limited settings. Children with sickle cell disease (SCD) are susceptible due to weakened immunity, influencing infection outcomes. This sub-study, nested within the Kenya Child Health and Mortality Prevention Surveillance (CHAMPS) network, investigated infectious pathogens associated with stillbirths and under-five deaths in Western Kenya, as well as the role of the sickle cell condition.

**Methods:**

We analysed stillbirths (*n* = 387) and ≤ 5 child deaths (*n* = 945) enrolled in CHAMPS in Karemo (Siaya) and Manyatta (Kisumu), both in western Kenya, between May 2017 and Dec 2024. Minimally invasive tissue sampling (MITS) specimens were processed on a TaqMan Array Card using the QuantStudio 7 real-time PCR (TAC qPCR) and cultured. All participants were screened for sickle cell status using the point-of-care Gazelle™ Hb Variant device, and confirmed by High Performance Liquid Chromatography (HPLC) and pathological diagnosis. Statistical analyses were performed using R programming software (version 4.5.1).

**Findings:**

A total of 1,332 cases were investigated, 499, 37.5% (95% CI:34.9–40.1) had an infectious pathogen (IP), with lower prevalence among stillbirths (4.1%, 95%CI:2.6–6.6) and higher prevalence among under-five deaths (51.1%, 95%CI:47.9–54.3). SCD was identified in 46 cases (3.5%) and sickle cell trait in 256 cases (19.2%), for a total of 302 cases (22.7%) with SCD/trait. IP prevalence was higher in SCD (60.9%, 95%CI: 46.3–73.9) than in sickle cell trait (25.8%, 95%CI: 20.6–31.6). Among SCD and sickle cell trait cases, the most frequently identified pathogens were *Plasmodium falciparum* (39.3% and 18.2%), *Klebsiella pneumoniae* (7.1% and 37.9%), and *Streptococcus pneumoniae* (21.4% and 9.1%), respectively. Overall, the leading pathogens identified in post-mortem specimens in both SCD/trait and non-SCD/trait groups were *K. pneumoniae* (28.7% vs. 25.5%), *P. falciparum* (24.5% vs. 35.9%), *S. pneumoniae* (12.8% vs. 12.1%), HIV (8.5% vs. 8.7%), *cytomegalovirus* (7.4% vs. 7.4%), and *respiratory syncytial virus* (3.2% vs. 1.5%).

**Conclusions:**

Infectious pathogens were significantly associated with stillbirth and under-five mortality in Western Kenya, highlighting the need to strengthen infection prevention, diagnosis, and management during pregnancy and early childhood in line with WHO recommendations and targeted preventive care for vulnerable populations like those with SCD.

**Clinical trial number:**

Not applicable.

**Supplementary Information:**

The online version contains supplementary material available at https://doi.org/10.1186/s12879-026-13848-9.

## Introduction

Under-five mortality remains a pressing global health challenge, with approximately 4.9 million children dying before their 5th birthday, according to WHO 2023 estimates. In low- and middle- income countries (LMICs), particularly across sub-Saharan Africa, a disproportionate burden accounting for nearly half of under-five deaths despite comprising of 14% of the world’s child population [1, 2]. In 2022, the total under-five mortality globally reduced from 76 deaths per 1000 live births in 2000 to 37 deaths per 1000 live births. However, sub-Saharan Africa still records a high under-five mortality rate of 70 deaths per 1000 live births, 10 times higher than the under-five mortality rate in European countries [2]. In Kenya, despite under-five mortality reducing from 115 deaths per 1000 live births in 2003 to 41 deaths per live birth in 2021, it is still significantly high [3].

The high death rates in resource-limited countries are mainly attributed to infections that cause severe sepsis, respiratory infections, malaria and diarrhoea, and complications that include pre-term births, asphyxia and congenital anomalies [4–6]. However, in LMICs, the contribution of specific infectious pathogens to these outcomes is often poorly characterised due to limitations in diagnostic capacity and incomplete post-mortem investigations. The reliance on verbal autopsy and symptom-based reporting underestimates the true burden of infection-related deaths, especially in rural Kenya, where facility-based laboratory capacity and standardised post-mortem microbiological examination are a challenge [7].

Sickle cell disease (SCD) is a group of inherited blood disorders associated with synthesising abnormal haemoglobin molecules, particularly haemoglobin-S (Hb-S), which causes erythrocytes to take up a sickle shape. This condition predominantly affects people from African, Mediterranean, Middle Eastern, and South Asian origins [8, 9]. Africa bears a disproportionately high burden of sickle cell disease and sickle cell trait, both of which are known to influence susceptibility to infection and disease severity, yet their role in pathogen-associated mortality has not been sufficiently examined [10]. In 2021, Sub-Saharan Africa (SSA) recorded the highest mortality cases directly linked to SCD, with approximately 30,000 deaths, representing a 30.1% increase from an estimated 2.3 million in 2000 to 7.8 million in 2021 [11]. In Kenya, about 20,000 to 30,000 babies are born with sickle cell disease per year, and approximately 50% to 80% of them die before their fifth birthday [12]. Roughly 17 per cent of children in the lake region have sickle cell traits, and 0.6 per cent of them have sickle cell disease, amounting to 0.9 per cent of the overall prevalence of SCD across the nation [12]. The predisposition of children living with SCD is brought about by immune incompetence resulting from auto-splenectomy and functional hypersplenism, making them susceptible to bacterial and viral infection [6, 11]. In low-resource countries, inadequate access to quality healthcare services and high costs of effective therapies like hydroxyurea and stem-cell transplantation exacerbate this vulnerability. The high mortality of SCD infants in Kenya is mainly attributed to inadequate healthcare facilities, inappropriate use of penicillin prophylaxis, and unavailability of vaccines in some medical centres [11].

Existing studies have primarily focused on general infectious pathogens in children, but few have examined the specific contribution of sickle cell conditions to deaths from infections. Lack of efficient mortality surveillance has led to underestimation and misinterpretation of infectious causes of death in existing studies. Child Health and Mortality Prevention Surveillance (CHAMPS) is a multisite mortality surveillance network that operates in sub-Saharan Africa and Southeast Asia, with the aim of tracking definitive causes of stillbirths and under-five deaths using a comprehensive MITS procedure.

This sub-study, nested within the Kenya CHAMPS network, aimed to characterise infectious pathogens associated with stillbirth and under-five mortality and to examine the effect of sickle cell disease condition (SCD/trait).

## Methods

**Fig. 1:**
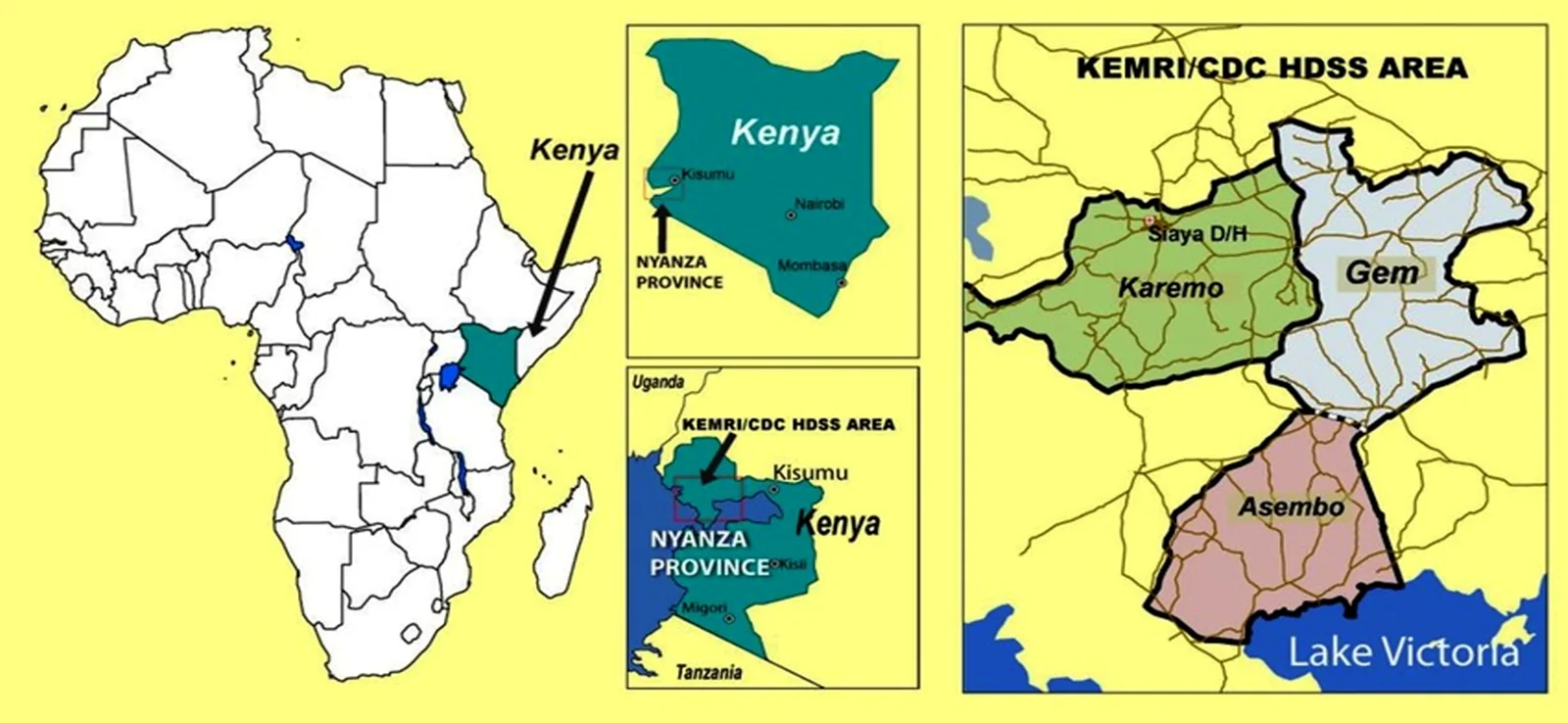
A map showing Health and Demographic Surveillance Systems (HDSS) in Western Kenya (https://www.champshealth.org/) [15]

**Fig. 2:**
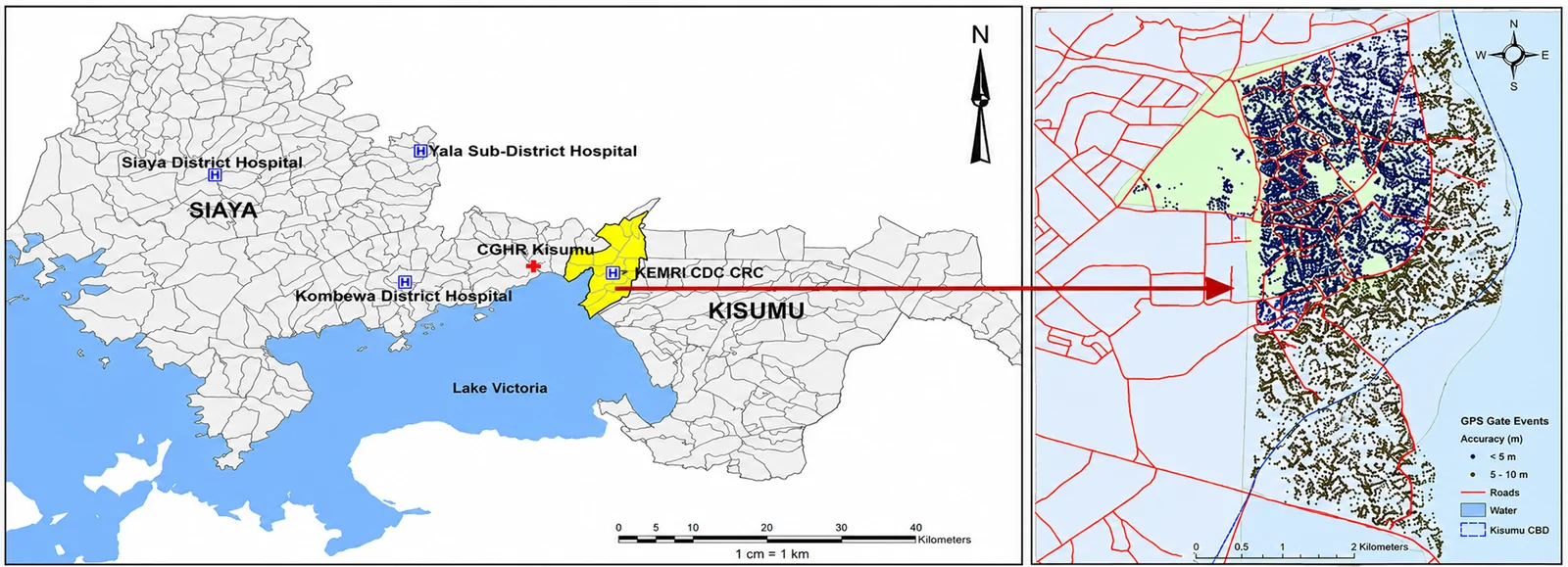
A map showing Karemo HDSS (Siaya County) and Manyatta HDSS (Kisumu County) (https://www.champshealth.org/) [15]

This study was a retrospective cross-sectional study utilising mortality surveillance data from the Kenya Child Health and Mortality Prevention Surveillance (CHAMPS) network. Kenya CHAMPS leverages two existing Health and Demographic Surveillance Systems (HDSS) in western Kenya: Karemo HDSS (Siaya County) and Manyatta HDSS (Kisumu County) [13, 14] (Figs. 1 and 2).

The catchment is characterised by high under-five mortality of 54 deaths per live birth [16, 17], a substantial infectious-disease burden of 59.9% [18], and a high prevalence of SCD of 0.6% [12, 19]. The study included all stillbirths and deaths among children aged ≤ 60 months enrolled in CHAMPS between May 2017 and December 2024, following the CHAMPS protocol and methods (https://www.champshealth.org/) [13, 20]. Mortality events were identified through community and facility notifications within the surveillance areas.

The study included deceased children under 60 months and stillbirths who met specific requirements as captured in the CHAMPS Protocol (https://www.champshealth.org/) [13, 19, 20].

Eligible decedents were those for whom the family consented, and post-mortem biological specimens were collected within 24 h for pathogen testing, and for whom SCD status could be confirmed through laboratory testing. A case with an infectious pathogen was defined as one in which one or more of the microorganisms tested for was identified. Stillbirths were defined as foetal deaths occurring at ≥ 28 weeks of gestation, or with birthweight ≥ 1000 g, or crown-heel length > 35 cm, with no sign of life, consistent with CHAMPS protocol. Enrolment required informed consent from a parent or guardian and availability of suitable post-mortem specimens for pathogen identification and SCD confirmation.

However, exclusion criteria were also based on CHAMPS Protocol guidelines [13, 20]. CHAMPS protocol excludes cases where consent was not obtained; bodies had been cremated or embalmed before sampling; or the death was subject to medicolegal investigation or regulatory procedures that precluded post-mortem sampling [13, 20]. These criteria were applied to preserve ethical standards, data integrity, and the reliability of laboratory analyses.

### Data collection methods

Data collection consisted of both clinical and laboratory procedures according to the CHAMPS Protocol (https://www.champshealth.org/). Clinical data such as demographic information, medical history, healthcare access patterns and co-morbidities were abstracted from medical records and verbal autopsy forms. Laboratory information was acquired through investigations of post-mortem biological specimens, including tissue and non-tissue sampling. These samples were processed to identify infectious pathogens using Multiplex PCR and microbiological procedures. The study included all infectious pathogens detected as either intermediate, antecedent or underlying in the cause of chain as confirmed by CHAMPS Determination of Cause of Death (DeCoDe) Panel Intermediate infectious pathogen was defined as a microbe that was in the causal chain that contributed to the development of subsequent diseases that led to stillbirth or under-five death, antecedent infectious pathogen was defined as a microbe that was identified in the cause of chain that contributed to development subsequent disease processes, while underlying pathogen was identified as a microbe that initiated the train of events or circumstance that lead to death. In addition, sickle cell status was determined using the point-of-care Gazelle™ Hb Variant device (Hemex Health) and confirmed by HPLC and by pathological diagnosis through the Determination of Cause of Death (DeCoDe) Panel.

### Sample collection

The samples were collected in a sterile environment using the standard Minimal Invasive Tissue Sampling (MITS) kits within 24 h, which consisted of biopsy needles (14 G-18 G), forceps, sterile gloves, scalpels and blood culture bottles as per CHAMPS methods [21]. The intended puncture sites of the body were externally disinfected with povidone-iodine. The blood samples were collected through percutaneous puncture of the heart and key vessels using a sterile syringe. Heart puncture was done through the left central thoracic region at the 5th intercostal space in a parasternal location. A sterile needle was inserted 5 cm in a sagittal direction while gentle aspiration and slight repositioning were done until blood was obtained, especially for stillbirth and neonate cases. Ten millilitres of blood were collected in paediatric blood culture bottles and EDTA tubes for microbiological examinations and TAC analysis [21]. Cerebrospinal fluid (CSF) was collected through lumbar puncture between the L3 and L5 vertebrae, providing 5 ml of fluid into sterile tubes [14, 22]. Tissue samples from the lungs, liver and brain were collected by penetrating biopsy needles through anatomic structures: the right upper quadrants for the liver, the left subcostal margins for the spleen, and the mid-clavicular line for the lungs. Brain tissues were collected in addition to nasopharyngeal and rectal swabs to investigate respiratory and gastrointestinal pathogens. All the samples were collected post-mortem from stillbirths and deceased under-five decedents.

### Laboratory procedures

#### Multiplex PCR

The study used TAC testing on the ViiA7 and Quant-Studio 7 (QS7) Real-Time PCR (qPCR) machines to identify various pathogen types from post-mortem samples. TAC is a multi-pathogen identification system comprising a 384-well array vessel used to amplify nucleic acid based on TaqMan real-time PCR technology [23, 24]. TAC wells contained dried primers and hydrolysis probes for the detection of targeted infectious pathogens or control targets. The amplification levels were corrected to the fluorescence data generated and measured using the QS7 Flex qPCR instrument, which indicated the presence of targeted nucleic acid in the sample [25]. The process for identification started with total nucleic acid extraction and was followed by TAC testing.

#### Total nucleic acid (TNA) extraction

Nucleic acid was extracted from post-mortem samples, including blood, lung tissues, brain tissues, cerebrospinal fluids (CSF), rectal swabs, oropharyngeal/ nasopharyngeal (OP/NP) swabs, using a standardised procedure to maximise yield and maintain the integrity across different samples. CSF and blood, Anticoagulant (EDTA) tubes were used to collect whole blood, and 400 µl was used in the extraction. 1 ml of phosphate-buffered saline (PBS) was used to suspend the rectal swab and vortexed; 400 µl of the specimen was utilised [25]. Respiratory swabs (OP/NP) were combined in universal transport medium, and 350 µl of the resultant specimen was used. 1 ml of bacteria lysis buffer (BLB) was used to soak lung tissues and then homogenised, maintaining a tissue to buffer ratio of 1:1. Infectious pathogens were inactivated in each sample by adding BLB, achieving a 1:1 ratio of buffer to specimen volume.

Furthermore, RNases and Dnases were inactivated by adding 25 mg/ml Protease K at a 10% volume ratio. Mechanical lysis steps differed based on the sample type: CSF, blood, and rectal swabs were disrupted by 0.5 mm glass beads to achieve a maximum disruption of difficult microbes to lyse; tissue homogenisation was done on lung tissues using 7 mm stainless steel beads. Mechanical lysis was not necessary for respiratory swabs. Thereafter, in the inactivation and lysis steps, a 700 µL volume of each prepared sample was collected for further processing. TNA was then extracted using an automated machine that ensured high-quality TNA suitable for subsequent TAC testing.

#### TAC testing on multiplex PCR

The study utilised CHAMPS customised TAC plates organised into four unique designs that consisted of under-five assays; CSF/blood tier 1 and tier 2 designs. The samples were tested on 4 sets of TACS that included enteric, CSF/blood tier 1, respiratory, and CSF/blood tier 2, which accommodated six test samples and two controls in every run to enhance data integrity and quality [25].

After the extraction of TNA, a reaction mixture was prepared by thawing qScript XLT 1-Step RT- qPCR Tough Mix (enzyme mix) and kept at 4 °C. No template controls (NTC) containing sterile nuclease-free water and positive controls (PC), consisting of synthetic nucleic acid templates, were set up. The reaction mixes were placed in a designated sterile area by combining 10 µl of extracted TNA and enzyme mix per run. The study used aerosol-resistant tips to pipette 100 µl of the mixture for each specimen and control, respectively, into TAC ports, preventing cross-contamination and air bubbles.

The TAC was then centrifuged twice at 1200 revolutions per minute for a minute each to enhance the even distribution of the mixture across the wells. The TAC was then trimmed off the fill reservoirs after being sealed with a mechanical sealer. The sealed cards were then inserted into the ViiA7 qPCR instrument for running. The setup of each run was based on template conditions appropriate for each TAC design. The run included amplification cycles, reverse transcription steps, and polymerase activation, adding up to 45 cycles. The amplification data were extracted and analysed using the QS qPCR Software v1.2, CDC. The threshold parameters were reviewed manually to distinguish between the real signal and background noise. A specimen was considered positive for the targeted infectious pathogen if a clear application curve crossed the expected cycle threshold (Ct) value between Ct 28–40, based on the target pathogen. Internal quality control of amplification, such as MS2 bacteriophage for stool samples and Rnase P for human samples, were examined for each specimen to ensure the absence of PCR inhibitors and nucleic acid integrity. Based on the amplification curves, the outputs were classified as negative, indeterminate or positive, and those runs that failed internal quality checks, like amplifications in negative control and unobservable amplification from positive controls, were discarded and repeated.

#### Bacterial culture

This study used two culture methods: an automated blood culture system using the BD Bactec FX40 Machine and a conventional microbial culture on culture media as per CHAMPS methods (https://www.champshealth.org/).

##### Automated blood culture

Blood collected in paediatric blood culture bottles from the field was immediately transported to the laboratory for incubation. These bottles contained media that supported the growth of a wide range of microorganisms, including both aerobic and anaerobic bacteria. The bottles were placed in the BD Bactec FX40 system, which was incubated and continuously monitored for microbial growth. The system uses fluorescence-based technology to detect changes in the carbon dioxide levels produced by microbial metabolism, indicating the presence of microorganisms [26]. The system detected most bacteraemia cases within a 5- to 7-day incubation period.

When microbial growth was detected, the system flagged the bottle as positive. The time to positivity (TTP) varied depending on the organism and the blood volume used. The system provided rapid detection, which was crucial for the timely diagnosis and further examination of bloodstream infections [27]. Positive cultures were subjected to further investigations, such as Gram staining and subculturing, to identify the specific microorganisms involved. This step was essential for guiding the appropriate antimicrobial panel [26].

##### Bacterial media culture

A positive blood culture from the Bactec machine was immediately processed for subculturing. The rubber septum of the bottles was aseptically disinfected using 70% Isopropyl alcohol. A sterile syringe and needle were then used to aspirate 5 ml of the blood broth mixture, which was inoculated on three primary media: Blood agar (BA), Chocolate agar (CHOC), and MacConkey agar (MAC). Blood agar was used to support a wide range of gram-negative microbes, and chocolate agar was incubated under 5–10% carbon dioxide conditions, which facilitated the growth of fastidious microorganisms like *Haemophilus influenzae* and Neisseria species. MacConkey agar was used to isolate gram-negative enteric bacteria and to differentiate non-lactose fermenters from lactose fermenters. The inoculated Petri dishes were incubated at 37°c with BA and MAC maintained at aerobic conditions, and CHOC incubated in a carbon dioxide-enriched environment. Plates were examined after 18–24 h, with the second inspection at 48 h of incubation to capture any slow-growing pathogens. Bacterial colonies were identified based on morphological features on the media plate and Gram-staining results.

For CSF specimens collected through MITS, 0.5 ml was inoculated directly onto BA, MAC and CHOC plates after reception in the laboratory. The same incubation protocol was followed as for blood culture, with CHOC plates maintained in a carbon dioxide environment. In addition to culture, a direct Gram stain technique was done on CSF to provide rapid preliminary results on morphology, presence and Gram stain status of any microbe. Incubated plates were examined after 24 and 48 h. Isolated microbes were identified using catalase and oxidase testing [28].

### Sickle cell testing

This study used the point-of-care Gazelle™ Hb Variant device (Hemex Health) and the HPLC (Bio-Rad D-10) to describe the haemoglobin variants of all the participants’ samples. A standardised procedure was followed as per the manufacturer’s instructions to ensure reproducibility and reliability of results. The blood samples collected during the MITS procedure were loaded into the Gazelle disposable cartridge. Each cartridge was prefilled with a separation buffer, and a 20 µl blood sample was carefully loaded into the application area. The cartridge was then loaded into a Gazelle Hb Variant testing machine, a portable microchip-based electrophoresis platform. The machine separated the haemoglobin variants in the applied blood samples on cellulose acetate paper housed in the cartridge. The devices relied on the haemoglobin electrophoresis principle, where Hb variants comprising of Haemoglobin Sickle C (Hb SC), haemoglobin S (Hb S), haemoglobin A (Hb A), haemoglobin A2 (Hb A2), haemoglobin F (Hb F), and haemoglobin E (Hb E) have distinguished net negative charge in alkaline solution [29]. This difference in negative charge allowed them to move across the paper at different speeds due to the applied voltage, separating Hb variants into visible bands on the paper. In approximately 8 min, the gazelle gave results that were visually represented as bands and quantitatively reported as the relative percentage of each haemoglobin type present.

### Data processing and analysis

Data collection and organisation were conducted using the KoBo Toolbox and Research Electronic Data Capture (REDCap) software, which provided reliability and security for managing field and clinical information. The data collected was cleaned and securely stored. Data system access was limited to a few authorised research personnel by password-supported accounts, and physical access to study material was restricted. Personal study identifiers were used to maintain participant confidentiality.

Statistical analyses were performed using R programming software (version 4.5.1). Descriptive statistics were used for demographic characteristics age and gender, types of infectious pathogens identified, and mortality trends. A comparative analysis assessed the differences and burdens of infectious pathogens between mortality cases with SCD/trait and those without SCD/trait. Pearson’s chi-square tests and Fisher’s exact tests were used to test for independence of association between SCD status and the infectious pathogen identified, with a significance level maintained at *p* < 0.05.

## Results

The findings are from 1332 cases, of which 387 were stillbirths and 945 were from under-five cases. The overall prevalence of IP in the study population was 37.5% (95% CI: 34.9%, 40.1%), in the stillbirth cases was 4.1% (95% CI: 2.6%, 6.6%), and in under-five death was 51.1% (95% CI: 47.9%, 54.3%). Of the 1332 cases, 46 (3.5%) had sickle cell disease, and 256 (19.2%) had sickle cell trait, totalling 302 (22.7%) with SCD/trait. The prevalence of IP in sickle cell disease was 60.9% (95% CI: 46.3%, 73.9%) and in sickle cell traits was 25.8% (95% CI: 20.6%, 31.6%), with top pathogens being *Plasmodium falciparum* 11cases (39.3%), 12 cases (18.2%), *Klebsiella pneumoniae* 2 cases (7.1%), 25 cases (37.9%), *Streptococcus pneumoniae*, 6 cases (21.4%), 6 cases (9.1%) respectively. Sex was determined in 1319 cases included in analysis. Findings on sex-specific analysis showed a balanced distribution, with females accounting for 46.5% of deaths among SCD/trait compared to 45.5% among non-SCD/trait, and males comprising 53.5% SCD/trait vs. 54.5% non-SCD/trait deaths. The observable differences were not statistically significant (*p* = 0.8) (Table 1).

The analysis of deaths stratified by SCD status showed notable variations across age categories (p = < 0.001). Among non-SCD/trait cases, stillbirths accounted for 29.8% of deaths as compared to 26.5% among those with SCD/traits. Contrary to deaths within the first 24 h of life, which were proportionally higher among SCD/trait group (21.5%) than in non-SCD/trait cases (12.0%). 1 day to less than 7 days old comprised 9.0% of non-SCD/trait deaths and 10.6% of SCD/trait deaths, while 7 days to 27 days old were relatively comparable (4.7% vs. 5.3%). In 28 days to less than 12 months old, 23.5% were accounted for by non-SCD/traits and 17.5% SCD/trait deaths. In 12 months to less than 60 months old categories, there were relatively higher non-SCD/trait death rates (21.0%) compared to SCD/trait deaths 18.5% (Table 1).

The majority of deaths occurred in health facilities (75.4% *n* = 1,004), while 24.6% (*n* = 328) occurred in the community. When stratified by group, a higher proportion of deaths among SCD/trait cases occurred in health facilities, 244 cases (80.8%), compared to non-SCD/trait 760 cases (73.8%). Conversely, community deaths were less frequent among SCD/trait cases (19.2%, *n* = 58) than non-SCD/trait 270 cases (26.2%). This difference in the distribution of location of death between groups was statistically significant (*p* = 0.013) (Table 1).

**Table 1:** Characteristics of stillbirths and deaths enrolled in the study

Characteristic	*N*	Overall	Non SCD/trait,	SCD/trait,	*p*-value^1^
		*N* = 1,332	*N* (%) = 1030 (77.3%)	*N* (%) = 302 (22.7%)	
Sickle Cell Disease type, n (%)	1,332				
Non SCD		1,030 (77.3%)			
Sickle Cell disease		46 (3.5%)		46 (15.2%)	
Sickle Cell trait		256 (19.2%)		256 (84.8%)	
Age at death, n (%)	1,332				< 0.001
Stillbirth		387 (29.1%)	307 (29.8%)	80 (26.5%)	
Death in the first 24 h		189 (14.2%)	124 (12.0%)	65 (21.5%)	
1 to 6 days old		125 (9.4%)	93 (9.0%)	32 (10.6%)	
7 to 27 days old		64 (4.8%)	48 (4.7%)	16 (5.3%)	
28 days old to < 12 months old		295 (22.1%)	242 (23.5%)	53 (17.5%)	
12 months old to < 60 months old		272 (20.4%)	216 (21.0%)	56 (18.5%)	
Catchment Area, n (%)	1,332				0.7
Karemo		679 (51.0%)	528 (51.3%)	151 (50.0%)	
Manyatta		653 (49.0%)	502 (48.7%)	151 (50.0%)	
Location of Death, n (%)	1,332				0.013
Community deaths		328 (24.6%)	270 (26.2%)	58 (19.2%)	
Health Facility deaths		1,004 (75.4%)	760 (73.8%)	244 (80.8%)	
Sex, n (%)	1,319				0.8
Female		603 (45.7%)	463 (45.5%)	140 (46.5%)	
Male		716 (54.3%)	555 (54.5%)	161 (53.5%)	

^1^ Pearson’s Chi-squared test; Fisher’s exact test, N - Proportions based on the number of cases

### Infectious pathogens identified

From the 1332 cases enrolled in the study, 499 had an infectious pathogen identified in their post-mortem samples, with a prevalence of 37.5% (95% CI: 34.9%, 40.1%). The distribution of infectious pathogens identified was as follows: bacteria accounted for the highest number of infections among the total cases, with 305,61.8% (95% CI 56.7%, 65.4%); parasites, 171, 34.3% (95% CI 30.1%, 38.6%); viruses, 118, 23.6% (95% CI 20.0%, 27,7%); and fungi, 9, 1.8% (95% CI 0.9%, 3.52%) (Table 2). Among 302 cases with SCD/trait enrolled in the study, pathogens were identified in 94 cases (31.1% [95% CI 26.0%, 36.7%]). Bacterial infections were the most prevalent, accounting for 60 out of 94 cases (63.8%). Viral infections were identified in 25 cases (26.6%), parasites in 23 cases (24.5%), and fungal pathogens were not identified in this group.

**Table 2:** Prevalence of infectious pathogens in the study population

Infectious Pathogen (IP)	*N*	Overall	Non SCD/trait,	SCD/trait,	*p*-value^1^
		*N* = 1,332	*N* (%) = 1030 (77.3%)	*N* (%) = 302 (22.7%)	
Infectious Conditions, n (%)	1,332				0.008
Infectious		823 (61.8%)	656 (63.7%)	167 (55.3%)	
Non-infectious		509 (38.2%)	374 (36.3%)	135 (44.7%)	
Infectious Pathogen Identified, n (%)	1,332	499 (37.5%)	405 (39.2%)	94 (31.1%)	0.011
Bacteria, n (%)	499	308 (61.8%)	248 (61.4%)	58 (61.7%)	0.9
Virus, n (%)	499	118 (23.6%)	90 (22.2%)	28 (26.6%)	0.12
Parasite, n (%)	499	171 (34.3%)	148 (36.6%)	23 (24.5%)	0.026
Fungi, n (%)	499	9 (1.9%)	9 (2.2%)		0.2

^1^ Pearson’s Chi-squared test; Fisher’s exact test, N - Proportions based on the number of cases

In children without SCD/trait, bacterial pathogens were also the most common, accounting for 248 of 405 cases (61.4%). Parasitic infections were identified in 148 cases (36.6%), viral infections in 88 cases (21.8%), and fungal pathogens in 9 cases (2.2%). The parasitic pathogens were less prevalent among SCD/trait cases (24.5%) than among non-SCD/trait cases (36.6%). The difference was statistically significant (*p* = 0.026) (Table 2).

**Table 3:** Distribution of infectious pathogens among cases with pathogen isolated (*N* = 499)

	Infectious Pathogens	Overall	No SCD/trait	SCD/trait	*p*-value^1^	q-value^2^
		*N* = 499	*N* = 405	*N* = 94		
Parasite	*Plasmodium falciparum*, n (%)	168 (33.7%)	145 (35.8%)	23 (24.5%)	0.048	0.6
	*Ascaris lumbricoides*, n (%)	2 (0.4%)	2 (0.5%)	0 (0.0%)	> 0.9	> 0.9
	*Plasmodium malariae*, n (%)	1 (0.2%)	1 (0.2%)	0 (0.0%)	> 0.9	> 0.9
Bacteria	*Klebsiella pneumoniae*, n (%)	130 (26.1%)	103 (25.4%)	27 (28.7%)	0.6	> 0.9
	*Streptococcus pneumoniae*, n (%)	61 (12.2%)	49 (12.1%)	12 (12.8%)	> 0.9	> 0.9
	*Escherichia coli*, n (%)	56 (11.2%)	50 (12.3%)	6 (6.4%)	0.14	> 0.9
	*Staphylococcus aureus*, n (%)	30 (6.0%)	26 (6.4%)	4 (4.3%)	0.6	> 0.9
	*Streptococcus spp.*, n (%*)*	17 (3.4%)	16 (4.0%)	1 (1.1%)	0.3	> 0.9
	*Haemophilus influenzae*, n (%)	16 (3.2%)	13 (3.2%)	3 (3.2%)	> 0.9	> 0.9
	*Pseudomonas aeruginosa*, n (%)	11 (2.2%)	10 (2.5%)	1 (1.1%)	0.7	> 0.9
	*Streptococcus pyogenes*, n (%)	11 (2.2%)	9 (2.2%)	2 (2.1%)	> 0.9	> 0.9
	*Acinetobacter baumannii*, n (%)	8 (1.6%)	5 (1.2%)	3 (3.2%)	0.4	> 0.9
	*Streptococcus agalactiae*, n (%)	8 (1.6%)	7 (1.7%)	1 (1.1%)	> 0.9	> 0.9
	*Aeromonas spp.*, n (%)	5 (1.0%)	5 (1.2%)	0 (0.0%)	0.6	> 0.9
	*Enterobacter cloacae*, n (%)	5 (1.0%)	4 (1.0%)	1 (1.1%)	> 0.9	> 0.9
	*Salmonella spp.*, n (%)	5 (1.0%)	4 (1.0%)	1 (1.1%)	> 0.9	> 0.9
	*Escherichia coli/shigella spp.*, n (%)	4 (0.8%)	4 (1.0%)	0 (0.0%)	0.7	> 0.9
	*Citrobacter freundii*, n (%)	3 (0.6%)	3 (0.7%)	0 (0.0%)	> 0.9	> 0.9
	*Klebsiella spp.*, n (%)	3 (0.6%)	2 (0.5%)	1 (1.1%)	> 0.9	> 0.9
Virus	HIV, n (%)	43 (8.6%)	35 (8.6%)	8 (8.5%)	> 0.9	> 0.9
	*Cytomegalovirus* (CMV), n (%)	37 (7.4%)	30 (7.4%)	7 (7.4%)	> 0.9	> 0.9
	*Adenovirus*, n (%)	18 (3.6%)	15 (3.7%)	3 (3.2%)	> 0.9	> 0.9
	*Respiratory syncytial virus* (RSV), n (%)	9 (1.8%)	6 (1.5%)	3 (3.2%)	0.5	> 0.9
	*Rotavirus a*,* n (%)*	6 (1.2%)	4 (1.0%)	2 (2.1%)	0.7	> 0.9
	*Rotavirus non-typable*, n (%)	6 (1.2%)	5 (1.2%)	1 (1.1%)	> 0.9	> 0.9
	*Human metapneumovirus* (HMPV), n *(%)*	3 (0.6%)	1 (0.2%)	2 (2.1%)	0.2	> 0.9
	*Norovirus gii*, n (%)	3 (0.6%)	3 (0.7%)	0 (0.0%)	> 0.9	> 0.9
	*Influenza a*, n (%)	2 (0.4%)	0 (0.0%)	2 (2.1%)	0.042	0.6
	*Influenza b*, n (%)	2 (0.4%)	1 (0.2%)	1 (1.1%)	0.8	> 0.9
	*Norovirus gi*, n (%)	2 (0.4%)	0 (0.0%)	2 (2.1%)	0.042	0.6
	*Parainfluenza virus type 1*, n (%)	2 (0.4%)	2 (0.5%)	0 (0.0%)	> 0.9	> 0.9
	*Parainfluenza virus type 3*, n (%)	2 (0.4%)	1 (0.2%)	1 (1.1%)	0.8	> 0.9
	*Parvovirus b19*, n (%)	2 (0.4%)	1 (0.2%)	1 (1.1%)	0.8	> 0.9
Fungi	*Pneumocystis jirovecii*, n (%)	6 (1.2%)	6 (1.5%)	0 (0.0%)	0.5	> 0.9
	*Candida albicans*, n (%)	1 (0.2%)	1 (0.2%)	0 (0.0%)	> 0.9	> 0.9
	*Yeast*, n (%)	1 (0.2%)	1 (0.2%)	0 (0.0%)	> 0.9	> 0.9
	*Yeast fungaemia*, n (%)	1 (0.2%)	1 (0.2%)	0 (0.0%)	> 0.9	> 0.9

^1^ 2-sample test for equality of proportions with continuity correction, Proportions based on the number of cases

^2^ False discovery rate correction for multiple testing

### Bacterial pathogens

The most commonly identified bacterial agent was *Klebsiella pneumoniae*, detected in 130 cases (26.1%) overall (Supplementary Table 2). Of these, 103 cases (25.5%) were isolated from non-SCD/trait deaths, while 27 cases (28.7%) were from participants with SCD/trait, showing a slightly higher proportion than in non-SCD/trait cases (*p* = 0.6) (Table 3).

*Streptococcus pneumoniae* was the second most prevalent identified bacterium, detected in 61 cases (12.2%). Among these, 49 cases (12.1%) were detected in non-SCD/trait participants and 12 cases (12.8%) in SCD/trait. *Escherichia coli* was identified in 56 cases (11.2%), with a markedly higher prevalence in non-SCD/trait participants (50 cases, 12.4%) compared to the SCD/trait group (6 cases, 6.4%) but was not statistically significant (*p* = 0.14). *Staphylococcus aureus* was detected in 30 cases (6.0%), consisting of 26 non-SCD/trait cases (6.4%) and 4 SCD/trait cases (4.3%). For *Streptococcus pyogenes*, 9 cases (2.2%) were in non-SCD/traits groups and 2 cases (2.1%) in SCD/trait groups. *Acinetobacter baumanii* was noticed in 5 non-SCD/trait cases (1.2%) and 3 SCD/trait cases (3.2%), showing a notably higher proportion among SCD/trait participants (*p* = 0.4) (Table 3).

### Fungal pathogens

Fungal pathogens were relatively uncommon, accounting for 9 (1.1%) cases of the total infection-related deaths. They were only identified in non-SCD/trait participants, and no case was detected in SCD/trait. *Pneumocystis jirovecii* was detected in 1.2% (6/499) of the overall cohort, with all cases occurring in individuals without SCD/trait (1.5%, 6/405). In contrast, *yeast fungaemia* and *Candida albicans* were detected in 1 case (0.2%) each (Table 3).

### Viral pathogens

Analysis of viral pathogens detected in the study population showed a distinct pattern based on sickle cell status (Table 3). The prevalence of major viruses, including HIV (8.7% vs. 8.5%) and *Cytomegalovirus* (7.4% vs. 7.4%), was comparable between individuals without and with SCD/traits. In contrast, significant differences were observed for specific respiratory and gastrointestinal viruses. The SCD/trait group exhibited markedly higher detection rates for *Human Metapneumovirus* (HMPV) (0.2% vs. 2.1%) and *Respiratory Syncytial Virus* (RSV) (1.5% vs. 3.2%). A similar trend was seen for *Norovirus GI*, which was detected exclusively in the SCD/trait group (2.1%), and Rotavirus A (1.0% vs. 2.1%). Other viruses, including *Adenovirus* and *Parainfluenza* types, showed minimal variation between groups.

### Parasitic pathogens

The predominant parasitic agent detected in this study was *Plasmodium falciparum*, the causative pathogen of the most severe form of malaria. It was identified in 33.7% (168/499) of the overall cohort, making it the most prevalent pathogen identified across all infectious agent classifications. Among non-SCD/trait cases, *Plasmodium falciparum* was detected in 35.9% (145/405), while 24.5% (23/94) were in the SCD/trait group.

Other parasitic infections were infrequently detected. *Ascaris lumbricoides* was identified in two individuals (0.4%), both in the group without SCD/trait. *Plasmodium malariae* was detected in one individual (0.2%), also in the group without SCD/trait.

### Distribution of infectious pathogens by age group among SCD/trait cases

**Fig. 3:**
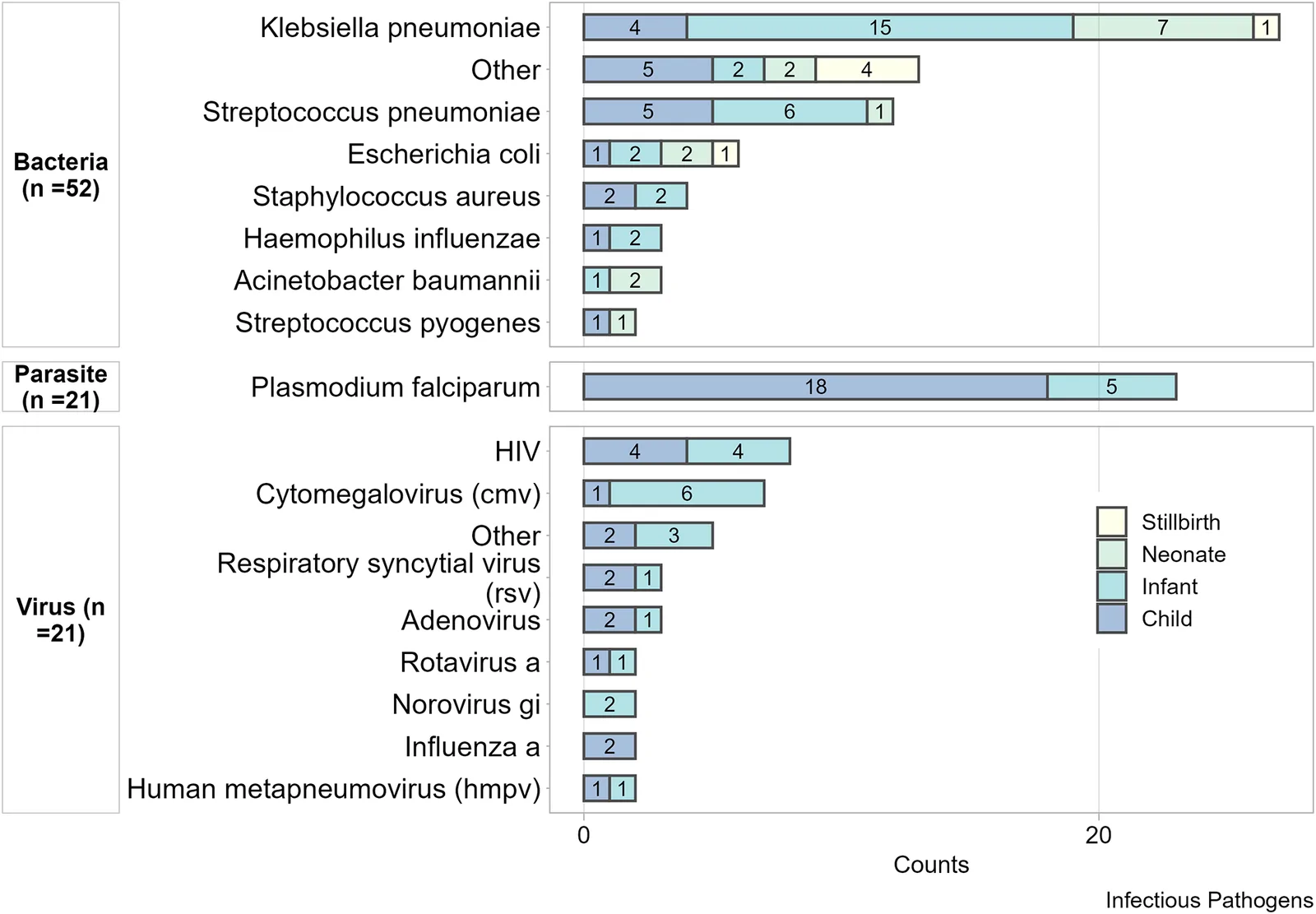
The distribution of infectious pathogens by age among SCD/trait cases

Figure 3 provides details on infectious pathogens across different age groups among SCD/trait cases (Supplementary Table 1). Among viral agents, CMV and HIV were most frequently identified, especially among infants and children. RSV and *Adenoviruses* were also prevalent, with moderate representation in infants and neonates. In addition, respiratory viruses such as *Influenza A*, *parainfluenza virus type 3*, and *Rotavirus A* were dominantly detected in infant groups. For parasitic infections, *Plasmodium falciparum*,* the* causative agent for malaria, was highly dominant, with the majority of deaths occurring in child and infants’ categories (Fig. 3).

Moreover, in bacterial infections, *Klebsiella pneumoniae* was the most predominant pathogen across all age groups, with a high burden observed among infants, followed by neonates and child categories (Fig. 3, Supplementary Table 1). *Streptococcus pneumoniae* was the second most prevalent bacterium detected, dominantly causing deaths in infants and children. *Staphylococcus aureus*,* Haemophilus influenzae and Acinetobacter baumannii* also appeared prominently, particularly in neonates and infants. *Escherichia coli* was detected in all the categories, including child, neonates, infants and stillbirths. (Fig. 3, Supplementary Tables 1 and 2).

## Discussion

The study aimed to characterise infectious pathogens associated with stillbirth and under-five deaths among decedents with SCD/trait in Western Kenya, and to compare their distribution with their counterparts without SCD/traits. The findings highlight infection as an important contributor to adverse pregnancy outcomes and child deaths, with distinct infectious pathogen profiles observed among SCD/trait cases. Among the infectious agents detected, *Plasmodium falciparum* was the most prevalent overall, accounting for 33.7% (168 cases). This insight shows a significant burden of malaria within the study setting. This underscores the continued public health concerns imposed by *Plasmodium falciparum* in endemic regions, especially in association with neonatal and maternal complications leading to stillbirths and mortality of the under-fives [30, 31].

Bacterial agents were predominantly presented in the findings, with *Klebsiella pneumoniae* 130 cases (26.1%), *Streptococcus pneumoniae* 61 cases (12.2%), and *Escherichia coli* 56 cases (11.2%) among the top three most prevalent bacterial pathogens in both groups with and without SCD/trait. These microbes are mainly associated with severe invasive infections like meningitis, pneumonia and sepsis, particularly in neonates and immunocompromised infants [32–34]. In addition, *Staphylococcus aureus*, *Haemophilus influenzae*, and *Streptococcus spp*. further reflects the diversity of bacterial agents implicated in children’s infections and stillbirths.

Viral agents also contributed significantly, with *Cytomegalovirus* (CMV) amounting to 7.4% (37 cases), and HIV 8.6% (43 cases) being the most frequently identified. These infections are particularly linked to potential vertical transmission and chronic complications [35, 36]. The insights detecting *Adenovirus* in 18 cases (3.6%) and *Respiratory Syncytial Virus* in 9 cases (1.8%) pointed out the role of respiratory viral infections in paediatric morbidity and mortality, especially in neonates and infants. Generally, the findings demonstrated a broad array of infectious pathogens contributing to stillbirths and deaths of children below 60 months, giving an overview of the complex interplay between host susceptibilities and environmental exposures.

### Infectious pathogens distribution (SCD/trait vs. non-SCD/trait)

The study findings on the distribution of infectious agents among stillbirths and under-five mortality cases with SCD/trait and those without showed notable differences in prevalence patterns. Reflecting on bacterial infections, *Klebsiella pneumoniae* was frequently detected in both groups, and was predominantly identified in the among SCD/trait cases (28.7%) compared to the non-SCD/trait group (25.5%); however, this difference was not statistically significant (*p* = 0.6) This higher infection may be associated with impaired immune responses and frequent hospitalisation in SCD/trait patients [37]. Similarly, *Streptococcus pneumoniae* revealed a slightly higher relative prevalence among SCD/trait cases (12.8%) than among those without SCD/trait (12.1%), but the difference was not statistically significant (*p* = 0.8) While previous studies have reported increased susceptibility to certain bacterial infections among individuals with sickle cell disease, particularly pneumococcal infections related to functional asplenia, our findings did not demonstrate statistically significant differences in pneumococcal pathogen prevalence by sickle cell status [6, 11, 38].

In Kenya, the National Guideline for the Prevention and Control of Common Childhood Illnesses stresses the importance of early diagnosis and management of febrile diseases in susceptible individuals, including SCD children [39]. However, our study shows continued vulnerability among this group, highlighting a policy-practice gap, especially in Western Kenya. Strengthening routine screening for bacterial and parasitic infections in SCD clinics could reduce preventable mortalities.

*Plasmodium falciparum* was identified as the leading parasitic agent and overall, the most prevalent. The high burden is attributable to the parasite’s propensity to cause severe disease manifestations such as cerebral malaria and severe anaemia, both of which are associated with high case fatality rates in young children [40]. *Plasmodium falciparum* infections exhibit distinct characteristics in patients with SCD and traits compared to those without. The study findings showed that children with SCD/traits were less likely to have malaria as a contributing factor to mortality compared to their counterparts without SCD/traits (*P* = 0.048). These findings support the existing knowledge that SCD/trait confers varying degrees of protection against malaria, primarily through mechanisms that affect parasite density and cytoadherence [41]. This protection is attributed to impaired cytoadherence of *Plasmodium falciparum*-infected erythrocytes containing sickle haemoglobin, reducing parasite sequestration in microvascular tissues [41]. Additionally, existing studies have indicated that HbAS is associated with a reduced risk of symptomatic malaria, although this protective effect can vary with age [42].

The World Health Organisation (WHO) recommends an integrated approach to malaria control, focusing on early diagnosis, effective treatment with artemisinin-based combination therapies, and preventive measures that include insecticide-treated nets and intermittent preventive treatment to pregnant women [43]. For high-risk groups such as children living with SCD, WHO guidelines indicate the need for intensified surveillance and tailored interventions, including regular screening and timely management of febrile illness [43]. The observed lower prevalence of severe malaria among SCD/trait patients aligns with existing studies for the partial protective effects of sickle haemoglobin (HbS), but it warrants the importance of maintaining comprehensive malaria prevention strategies in this vulnerable population. This ensures both effective prevention and management of malaria-related morbidity and mortality in vulnerable individuals.

This study reveals a distinct pattern of viral pathogen detection among individuals with SCD/trait compared to those without. While the prevalence of major viruses like HIV and CMV was nearly identical between groups, a markedly higher detection rate of specific respiratory and gastrointestinal viruses, namely *Human Metapneumovirus* (HMPV), *Respiratory Syncytial Virus* (RSV), *Norovirus GI*, and *Rotavirus A*, in the SCD/trait cohort. These findings show that an immunologic landscape of sickle cell conditions may confer a unique susceptibility to particular acute viral infections, even as it may leave responses to other persistent viruses unaffected. This slightly higher prevalence of HIV in the SCD/trait group may be due to their increased exposure to parenteral interventions such as blood transfusions and an underlying immunocompromised state. *Rotavirus A* accounted for 1.2% of cases, with a slightly higher prevalence in SCD/traits groups (2.1%) compared to non-SCD/traits groups (1.0%), which may indicate an increased predisposition due to impaired mucosal immunity. Notably, there was a higher prevalence of RSV infections in SCD/trait cases (3.2%) compared to the non-SCD/trait group (1.8%). This insight supports previous studies on the vulnerability of SCD patients to respiratory viral infections, due to chronic anaemia and functional asplenia [6, 11].

#### Distribution of infectious pathogens by age group among SCD/trait cases

The findings from a comprehensive analysis of the distribution of infectious pathogens by age among SCD/trait cases showed a diverse microbial landscape that contributes to stillbirth and mortality of children under 60 months. *Streptococcus pneumoniae* and *Haemophilus influenzae*, microbes specifically targeted by penicillin prophylaxis and Hib vaccines, were more prominent, particularly in the infant and child categories. These insights reinforce current pieces of evidence that individuals with SCD/trait have a high risk of invasive bacterial infections, particularly in early life [44]. These results are relevant in LMICs, especially in Kenya, where routine childhood immunisation programs, including the pneumococcal conjugate and Hib vaccines, are essential components of the Expanded Program of Immunisation (EPI). The continuous detection of these infectious pathogens may be attributed to suboptimal coverage of the vaccine, waning immunity, and the presence of non-vaccine serotypes in the population.

*Klebsiella pneumoniae* was the most prevalent bacterium detected across all age groups, including a notable number of infant and neonate deaths. This may be due to maternal colonisation and subsequent vertical transmission, and nosocomial acquisition in the perinatal care setting. The high burden of *Acinetobacter baumanii* and *Staphylococcus aureus* among SCD/traits, both known to be linked with hospital-acquired infections, especially in neonatal units, further supports the contribution of hospital-acquired infections in the neonatal deaths [45, 46]. The detection of *Plasmodium falciparum* across infants, children and neonates highlights the importance of malaria prevention strategies in SCD/trait individuals. Malaria is a known trigger for severe complications in SCD/trait individuals, including haemolysis, anaemia and increased mortality risks [47]. This data supports existing studies indicating that SCD children in malaria-endemic regions need targeted chemoprophylaxis and urgent treatment protocols [48]. Furthermore, CMV, HIV, and RSV were predominantly detected in children’s and infants’ samples. This dominance of CMV in infants may be linked to either congenital infection or reactivation in immunocompromised hosts. RSV was among the top detected viral pathogens in children and infants’ groups; its death-related cases are associated with its role in severe lower respiratory tract infections in early childhood.

The study for analytical purposes, individuals with Sickle Cell Disease and Sickle Cell Trait were combined into a single category (SCD/trait). This approach was adopted because the infectious pathogen profiles were comparable across the groups, with no evidence of meaningful differences observed in the analysis. However, this may obscure biologically meaningful differences, particularly given the distinct clinical and immunological characteristics of SCD and sickle cell trait. In addition, the study used a cross-sectional design and mortality data that do not allow for causal inference.

## Conclusions

The findings of the study showed that infectious pathogens remain a significant contributor to both stillbirths and deaths of children aged 1–59 months, particularly among vulnerable groups like those with SCD/traits. While many of the detected pathogens, *Plasmodium falciparum*,* Klebsiella pneumoniae* and *Streptococcus pneumoniae*, align with regional epidemiological trends, a few notable patterns emerged.

The study demonstrates that infectious pathogens continue to drive preventable stillbirths and under-five deaths, with sickle cell disease and trait further increasing vulnerability. Recommendations include rigorous adherence to WHO management guidelines for pregnant women with SCD or trait, enhanced screening and treatment of infectious agents, and systematic post-mortem investigation of deaths to clarify causality. Addressing these combined risks is essential to reducing mortality and improving maternal and child health outcomes in Kenya and similar LMIC contexts.

Furthermore, this study provides new insights into the pathogen-specific vulnerability of SCD/traits individuals, highlighting the complex interaction between host factors and infectious pathogens. By identifying the most prevalent pathogens associated with foetal outcomes in this population, the study offers practical knowledge to inform clinical prioritisation, guide critical treatment choices and shape targeted prevention strategies.

## Supplementary Information

Below is the link to the electronic supplementary material.

Supplementary Material 1

Supplementary Material 2

## Data Availability

Additional materials are available on the CHAMPS website (https://www.champshealth.org/) and upon reasonable request.

## Abbreviations

CHAMPS: Child Health and Mortality Prevention Surveillance
CI: Confidence Interval
DNA: Deoxyribonucleic Acid
KEMRI: Kenya Medical Research Institute
MITS: Minimal Invasive Tissue Sampling
NTC: No Template Control
PC: Positive Control
PCR: Polymerase Chain Reaction
RNA: Ribonucleic Acid
SCD: Sickle Cell Disease
SCT: Sickle Cell Trait
TAC: TaqMan Array Cards
UCC: University of Cape Coast
