# Supplementary material for: Infectious pathogens associated with stillbirth and under-five mortality by sickle cell disease in Western Kenya: a cross-sectional study
Source: BMC Infect Dis. 2026 Jun 30;26:1681. doi: 10.1186/s12879-026-13848-9 (PMC13589390; doi:10.1186/s12879-026-13848-9)
Supplement: Supplementary file 1 [file 12879_2026_13848_MOESM1_ESM.docx]

| **Supplementary Table 1: Comparison between Decedents with SCD/trait vs non-SCD/trait, by age group** | | | | | | | | | | |
| --- | --- | --- | --- | --- | --- | --- | --- | --- | --- | --- |
| **Characteristic** | **Non SCD/trait** | | | | | **SCD/trait** | | | | |
|  | **Overall** | **Stillbirth** | **Neonate** | **Infant** | **Child** | **Overall** | **Stillbirth** | **Neonate** | **Infant** | **Child** |
|  | N = 405*^1^* | N = 10*^1^* | N = 75*^1^* | N = 161*^1^* | N = 159*^1^* | N = 94*^1^* | N = 6*^1^* | N = 12*^1^* | N = 34*^1^* | N = 42*^1^* |
| *Plasmodium falciparum* | 145 (35.8%) | 2 (20.0%) | 2 (2.7%) | 51 (31.7%) | 90 (56.6%) | 23 (24.5%) |  |  | 5 (14.7%) | 18 (42.9%) |
| *Klebsiella pneumoniae* | 103 (25.4%) | 1 (10.0%) | 37 (49.3%) | 40 (24.8%) | 25 (15.7%) | 27 (28.7%) | 1 (16.7%) | 7 (58.3%) | 15 (44.1%) | 4 (9.5%) |
| *Escherichia coli* | 50 (12.3%) | 2 (20.0%) | 9 (12.0%) | 18 (11.2%) | 21 (13.2%) | 6 (6.4%) | 1 (16.7%) | 2 (16.7%) | 2 (5.9%) | 1 (2.4%) |
| *Streptococcus pneumoniae* | 49 (12.1%) |  | 6 (8.0%) | 22 (13.7%) | 21 (13.2%) | 12 (12.8%) |  | 1 (8.3%) | 6 (17.6%) | 5 (11.9%) |
| *HIV* | 35 (8.6%) |  |  | 12 (7.5%) | 23 (14.5%) | 8 (8.5%) |  |  | 4 (11.8%) | 4 (9.5%) |
| *Cytomegalovirus (*CMV*)* | 30 (7.4%) | 2 (20.0%) | 1 (1.3%) | 22 (13.7%) | 5 (3.1%) | 7 (7.4%) |  |  | 6 (17.6%) | 1 (2.4%) |
| *Staphylococcus aureus* | 26 (6.4%) | 1 (10.0%) | 5 (6.7%) | 13 (8.1%) | 7 (4.4%) | 4 (4.3%) |  |  | 2 (5.9%) | 2 (4.8%) |
| *Streptococcus spp.* | 16 (4.0%) | 2 (20.0%) | 2 (2.7%) | 7 (4.3%) | 5 (3.1%) | 1 (1.1%) |  | 1 (8.3%) |  |  |
| *Adenovirus* | 15 (3.7%) |  |  | 9 (5.6%) | 6 (3.8%) | 3 (3.2%) |  |  | 1 (2.9%) | 2 (4.8%) |
| *Haemophilus influenzae* | 13 (3.2%) |  |  | 5 (3.1%) | 8 (5.0%) | 3 (3.2%) |  |  | 2 (5.9%) | 1 (2.4%) |
| *Pseudomonas aeruginosa* | 10 (2.5%) |  | 3 (4.0%) | 4 (2.5%) | 3 (1.9%) | 1 (1.1%) |  |  |  | 1 (2.4%) |
| *Streptococcus pyogenes* | 9 (2.2%) |  | 2 (2.7%) | 2 (1.2%) | 5 (3.1%) | 2 (2.1%) |  | 1 (8.3%) |  | 1 (2.4%) |
| *Streptococcus agalactiae* | 7 (1.7%) | 1 (10.0%) | 4 (5.3%) | 2 (1.2%) |  | 1 (1.1%) | 1 (16.7%) |  |  |  |
| *Respiratory syncytial virus (*RSV*)* | 6 (1.5%) |  | 2 (2.7%) | 3 (1.9%) | 1 (0.6%) | 3 (3.2%) |  |  | 1 (2.9%) | 2 (4.8%) |
| *Pneumocystis jirovecii* | 6 (1.5%) |  |  | 5 (3.1%) | 1 (0.6%) |  |  |  |  |  |
| *Acinetobacter baumannii* | 5 (1.2%) |  | 3 (4.0%) | 1 (0.6%) | 1 (0.6%) | 3 (3.2%) |  | 2 (16.7%) | 1 (2.9%) |  |
| *Rotavirus non-typable* | 5 (1.2%) |  |  | 4 (2.5%) | 1 (0.6%) | 1 (1.1%) |  |  | 1 (2.9%) |  |
| *Aeromonas spp.* | 5 (1.2%) |  |  | 2 (1.2%) | 3 (1.9%) |  |  |  |  |  |
| *Enterobacter cloacae* | 4 (1.0%) |  | 4 (5.3%) |  |  | 1 (1.1%) |  |  | 1 (2.9%) |  |
| *Escherichia coli/shigella spp.* | 4 (1.0%) |  |  | 3 (1.9%) | 1 (0.6%) |  |  |  |  |  |
| *Salmonella spp.* | 4 (1.0%) |  | 1 (1.3%) | 1 (0.6%) | 2 (1.3%) | 1 (1.1%) |  |  |  | 1 (2.4%) |
| *Rotavirus a* | 4 (1.0%) |  |  | 3 (1.9%) | 1 (0.6%) | 2 (2.1%) |  |  | 1 (2.9%) | 1 (2.4%) |
| *Citrobacter freundi* | 3 (0.7%) |  | 2 (2.7%) | 1 (0.6%) |  |  |  |  |  |  |
| *Norovirus gii* | 3 (0.7%) |  |  | 3 (1.9%) |  |  |  |  |  |  |
| *Streptococcus species* | 2 (0.5%) |  |  |  | 2 (1.3%) |  |  |  |  |  |
| *Campylobacter jejuni* | 2 (0.5%) |  |  | 2 (1.2%) |  |  |  |  |  |  |
| *Haemophilus influenzae type a* | 2 (0.5%) |  |  | 2 (1.2%) |  |  |  |  |  |  |
| *Klebsiella spp.* | 2 (0.5%) |  |  | 1 (0.6%) | 1 (0.6%) | 1 (1.1%) |  |  |  | 1 (2.4%) |
| *Parainfluenza virus type 1* | 2 (0.5%) |  |  | 2 (1.2%) |  |  |  |  |  |  |
| *Enterococcus spp.* | 2 (0.5%) |  | 1 (1.3%) |  | 1 (0.6%) |  |  |  |  |  |
| *Proteus mirabilis* | 2 (0.5%) |  |  | 1 (0.6%) | 1 (0.6%) |  |  |  |  |  |
| *Ascaris lumbricoides* | 2 (0.5%) |  |  |  | 2 (1.3%) |  |  |  |  |  |
| *Enterococcus faecalis* | 2 (0.5%) |  |  | 2 (1.2%) |  |  |  |  |  |  |
| *Parainfluenza virus type 2* | 1 (0.2%) |  |  | 1 (0.6%) |  |  |  |  |  |  |
| *B. pertussis* | 1 (0.2%) |  |  | 1 (0.6%) |  |  |  |  |  |  |
| *Citrobacter sedlakii* | 1 (0.2%) |  | 1 (1.3%) |  |  |  |  |  |  |  |
| *Acinetobacter spp.* | 1 (0.2%) |  | 1 (1.3%) |  |  |  |  |  |  |  |
| *Enterococcus species* | 1 (0.2%) |  |  | 1 (0.6%) |  |  |  |  |  |  |
| *Haemophilus parainfluenzae* | 1 (0.2%) |  |  | 1 (0.6%) |  |  |  |  |  |  |
| *Influenza b* | 1 (0.2%) |  |  | 1 (0.6%) |  | 1 (1.1%) |  |  | 1 (2.9%) |  |
| *Serratia odorifera* | 1 (0.2%) |  |  | 1 (0.6%) |  |  |  |  |  |  |
| *Staphylococcus epidermidis* | 1 (0.2%) |  |  | 1 (0.6%) |  |  |  |  |  |  |
| *Yeast* | 1 (0.2%) |  |  | 1 (0.6%) |  |  |  |  |  |  |
| *Covid 19* | 1 (0.2%) |  | 1 (1.3%) |  |  |  |  |  |  |  |
| *Serratia spp* | 1 (0.2%) |  |  | 1 (0.6%) |  |  |  |  |  |  |
| *Parainfluenza virus type 4* | 1 (0.2%) |  |  | 1 (0.6%) |  |  |  |  |  |  |
| *Candida albicans* | 1 (0.2%) |  |  | 1 (0.6%) |  |  |  |  |  |  |
| *Serratia marcescens* | 1 (0.2%) |  |  |  | 1 (0.6%) |  |  |  |  |  |
| *Plasmodium malariae* | 1 (0.2%) |  |  |  | 1 (0.6%) |  |  |  |  |  |
| *Moraxella catarrhalis* | 1 (0.2%) |  |  | 1 (0.6%) |  |  |  |  |  |  |
| *Parainfluenza virus type 3* | 1 (0.2%) |  |  |  | 1 (0.6%) | 1 (1.1%) |  |  | 1 (2.9%) |  |
| *Rota virus* | 1 (0.2%) |  |  | 1 (0.6%) |  |  |  |  |  |  |
| *Mycobacterium tuberculosis* | 1 (0.2%) |  |  |  | 1 (0.6%) |  |  |  |  |  |
| *Human metapneumovirus (*HMPV*)* | 1 (0.2%) |  | 1 (1.3%) |  |  | 2 (2.1%) |  |  | 1 (2.9%) | 1 (2.4%) |
| *Staphylococcus spp.* | 1 (0.2%) |  |  |  | 1 (0.6%) | 1 (1.1%) |  | 1 (8.3%) |  |  |
| *Proteus vulgaris* | 1 (0.2%) |  |  |  | 1 (0.6%) |  |  |  |  |  |
| *Ureaplasma spp.* | 1 (0.2%) |  | 1 (1.3%) |  |  | 1 (1.1%) | 1 (16.7%) |  |  |  |
| *Chlamydia trachomatis* | 1 (0.2%) |  | 1 (1.3%) |  |  |  |  |  |  |  |
| *Bordetella spp.* | 1 (0.2%) |  | 1 (1.3%) |  |  |  |  |  |  |  |
| *Parvovirus b19* | 1 (0.2%) |  | 1 (1.3%) |  |  | 1 (1.1%) |  |  |  | 1 (2.4%) |
| *Aeromonas hypdrophila* | 1 (0.2%) |  |  |  | 1 (0.6%) |  |  |  |  |  |
| *Yeast fungaemia* | 1 (0.2%) |  | 1 (1.3%) |  |  |  |  |  |  |  |
| *Group c salmonella non-typhoid* | 1 (0.2%) |  |  |  | 1 (0.6%) |  |  |  |  |  |
| *Norovirus gi* |  |  |  |  |  | 2 (2.1%) |  |  | 2 (5.9%) |  |
| *Influenza a* |  |  |  |  |  | 2 (2.1%) |  |  |  | 2 (4.8%) |
| *Treponema pallidum* |  |  |  |  |  | 1 (1.1%) |  |  | 1 (2.9%) |  |
| *Ureaplasma urealyticum* |  |  |  |  |  | 1 (1.1%) | 1 (16.7%) |  |  |  |
| *Sapovirus i/ii/iv* |  |  |  |  |  | 1 (1.1%) |  |  |  | 1 (2.4%) |
| *Salmonella enterica sub-species typhi* |  |  |  |  |  | 1 (1.1%) |  |  |  | 1 (2.4%) |
| *Coagulase negative staphylococcus* |  |  |  |  |  | 1 (1.1%) | 1 (16.7%) |  |  |  |
| *Raoultella ornithinolytica* |  |  |  |  |  | 1 (1.1%) |  |  |  | 1 (2.4%) |
| *^1^* n (%) | | | | | | | | | | |
